# Culture and cannabinoid receptor gene polymorphism interact to influence the perception of happiness

**DOI:** 10.1371/journal.pone.0209552

**Published:** 2018-12-21

**Authors:** Masahiro Matsunaga, Takahiko Masuda, Keiko Ishii, Yohsuke Ohtsubo, Yasuki Noguchi, Misaki Ochi, Hidenori Yamasue

**Affiliations:** 1 Department of Health and Psychosocial Medicine, Aichi Medical University School of Medicine, Nagakute, Aichi, Japan; 2 Department of Psychology, University of Alberta, Edmonton, Alberta, Canada; 3 Department of Cognitive and Psychological Sciences, Graduate School of Informatics, Nagoya University, Nagoya, Aichi, Japan; 4 Department of Psychology, Graduate School of Humanities, Kobe University, Kobe, Hyogo, Japan; 5 Department of Psychiatry, Hamamatsu University School of Medicine, Hamamatsu, Shizuoka, Japan; Boston University, UNITED STATES

## Abstract

Previous studies have shown that a cytosine (C) to thymine (T) single nucleotide polymorphism (SNP) of the human cannabinoid receptor 1 (*CNR1*) gene is associated with positive emotional processing. C allele carriers are more sensitive to positive emotional stimuli including happiness. The effects of several gene polymorphisms related to sensitivity to emotional stimuli, such as that in the serotonin transporter gene-linked polymorphic region (5HTTLPR), on emotional processing have been reported to differ among cultures–e.g., between those that are independent and interdependent. Thus, we postulated that the effects of the *CNR1* genotype on happiness might differ among different cultures because the concept of happiness varies by culture. We recruited healthy male and female young adults in Japan, where favorable external circumstances determine the concept of happiness, and Canada, where the concept of happiness centers on positive inner feelings, and compared the effects of the *CNR1* genotype on both subjective happiness levels (self-evaluation as being a happy person) and situation-specific happiness (happy feelings accompanying various positive events) by using a questionnaire. We found that the effect of *CNR1* on subjective happiness was different between the Japanese and Canadian groups. The subjective happiness level was the highest in Japanese individuals with the CC genotype, whereas in Canadian participants, it was the highest in individuals with the TT genotype. Furthermore, the effects of *CNR1* genotype on situation-specific happiness were also different between the groups. Happiness accompanied with being surrounded by happy people was the highest among Japanese individuals with the CC genotype, whereas among Canadian individuals, it was the highest in TT genotype carriers. These findings suggest that culture and *CNR1* polymorphism interact to influence the perception of happiness.

## Introduction

The endocannabinoid system is a group of neuromodulatory lipids and their receptors [1]. The endocannabinoids, such as anandamide and 2-arachidonoyl glycerol [2–4], bind to brain cannabinoid receptors, which are involved in several physiological processes including appetite regulation, nociception, memory, and emotional processing [1–7]. Recent studies have indicated that a cytosine (C) to thymine (T) single nucleotide polymorphism (SNP) of the human cannabinoid receptor 1 (*CNR1*) gene (dbSNP number rs806377) is associated with positive emotional processing [5–7]. Studies have shown that the activity of the striatum, which is a part of the brain reward system, is higher, and gaze duration for faces is longer in C allele carriers than in individuals with the TT genotype when they are presented with a happy face [5,6]. These findings suggest that *CNR1* polymorphism may be strongly associated with sensitivity to external positive emotional stimuli, especially those related to human relationships. Furthermore, *CNR1* polymorphism has been associated with happiness [7]. Happiness is usually measured from two aspects: event-related temporal positive feelings (situation-specific happiness) and a relatively stable self-evaluation as being a happy person (subjective happiness level). These two aspects of happiness are interactive: those who cognitively evaluate themselves to be happy are more likely to experience happy events; conversely, those who frequently experience happy events are more likely to cognitively evaluate themselves as happy [8]. A previous study indicated that both the subjective happiness level and situation-specific happiness were higher in C allele carriers than in individuals with the TT genotype [7].

Previous studies have indicated that the concept of happiness varies by culture [9]. One such conception recognized in the United States, Canada, Spain, Argentina, and Ecuador among many other countries considers positive inner feelings, such as pleasure and joy, to be pivotal to obtaining happiness [9]. In contrast, happiness is generally recognized in Japan, Germany, Russia, Norwegian, and numerous other countries to be based on the fortune of external circumstances [10]: in response to being asked how happy they have been lately, such people would weigh their recent luck when they answer [9]. From these observations, a question arises: Is the effect of *CNR1* polymorphism on happiness common to all countries? Past research suggests that the allelic frequency of the serotonin transporter gene-linked polymorphic region (5HTTLPR), known to impact the expression and function of serotonin transporters [11,12], is associated with cultural values of individualism and collectivism [13]. Chiao and Blizinsky [14] indicated that collectivistic cultures were more likely to comprise individuals with short 5HTTLPR polymorphisms (S allele carriers), and a higher population frequency of the S allele predicted decreased anxiety and mood disorder incidences. Although 5HTTLPR S allele carriers may be more likely to be sensitive to emotional stimuli than individuals with long polymorphisms (L allele carriers) [11,12], the former may also be more adaptive in cultures oriented toward interdependence, leading to lower levels of mental disorders. Similarly, other gene polymorphisms, such as those of the serotonin and dopamine receptors, have also been implicated in the gene-culture interaction [15,16].

Based on these studies, it is possible that the effects of *CNR1* genotypes on happiness may differ across cultures that vary in their definitions of happiness. The level of subjective happiness in Japan was found to be higher in C-allele carriers than in individuals with the TT genotype [7]. The high level of subjective happiness among *CNR1-*C-allele carriers in Japan may be accounted for by a heightened sensitivity of *CNR1-*C-allele carriers to external positive emotional stimuli, rendering them well-adapted to the concept of happiness in Japanese culture. In contrast, it is possible that the subjective-happiness levels among *CNR1-*C-allele carriers is lower than that among individuals with the TT genotype in cultures where inner feelings inform happiness; a possible predisposition to inward-focused attention renders individuals with the TT genotype well-suited to cultures that emphasize individualism, while the high sensitivity to the positive states of others exhibited by C-allele carriers and the consequent social comparison in which they engage may diminish their subjective happiness in such cultures. Indeed, comparing one’s state with that of socially superior individuals can decrease one’s self-evaluation [17]. Of course, social comparison can also occur in Japan: previous studies have shown that Japanese people felt pain upon learning of the success of others–i.e., envy–[18] and people in collectivistic countries (including Japan) are more likely to be affected by social comparison relative to those in individualistic countries [19,20]. However, previous studies have indicated that people in collectivistic countries are more prone to emotional contagion than are people in individualistic countries [21,22]. The happiness of others may be more easily integrated into personal happiness in a collectivistic culture rather than in an individualistic one because people in collectivistic countries have been shown to have higher empathic abilities, associated with the social sharing of happiness [23–26], relative to those in individualistic countries [27]. The C-allele carries in Japan are therefore considered to be happier than those with the TT genotype. On the other hand, we speculate that the association between CNR1 genotypes and happiness may be reversed in Canada.

The present study therefore explores the hypothesis that subjective happiness levels in Japan are higher among *CNR1-*C-allele carriers than individuals with the TT genotype. Conversely, we further speculate that the subjective happiness levels in North American culture are lower among C-allele-carriers than individuals with the TT genotype. To test our hypothesis, we recruited university students from both Japan and Canada. First, in order to clarify differences in the perception of happiness, we compared subjective happiness levels and situation-specific happiness between Japanese and Canadian groups, and also conducted the correlation analyses to reveal the association between subjective happiness levels and situational happiness in both countries. Subsequently, we compared the effects of *CNR1* genotypes on subjective happiness levels and situation-specific happiness between the Japanese and Canadian groups in a questionnaire-based experiment.

## Materials and methods

### Participants

Based on previous studies indicating gene-culture interactions [14–16], we recruited 259 healthy Japanese volunteers (mean age: 19.51 years; range: 18−28 years; 102 males, 157 females) and 181 European Canadian volunteers (mean age: 19.47 years; range: 17−28 years; 59 males, 122 females) following the study’s approval by the Ethics Committees at Kobe University (approval number: 2014–10), Aichi Medical University (approval number: 14–036), and the University of Alberta (approval number: Pro00059940). All participants provided written informed consent in accordance with the Declaration of Helsinki. Participants were recruited from psychology subject pools at Kobe University, Aichi Medical University, and the University of Alberta. No participant was taking psychotropic drugs during the study. To eliminate possible confounding effects of sex, age, and psychiatric diseases on happiness [28,29], we focused on age-matched university students. Although they did not comprise representative samples of the healthy, young Japanese and Canadian populations, they did exhibit reasonable levels of variance in happiness. Therefore, the analyses in the present study were deemed unlikely to be affected by the range restriction problem.

### Evaluation of subjective happiness levels

To assess subjective happiness levels, participants completed the Subjective Happiness Scale (SHS) questionnaire [30,31]. The SHS exhibits excellent psychometric properties, such as high internal consistency, a unitary structure, and stability over time [30]. Therefore, the SHS is a widely used psychometric tool for evaluating subjective happiness levels [7,8]. The SHS subjectively assesses whether a person is happy or unhappy, as well as his or her positive personal traits. Each item is answered on a 7-point Likert scale. We asked participants to circle the point on the scale that they felt described them the most accurately. The items were as follows: 1 (general happiness): “In general, I consider myself…” 1 (not a very happy person) to 7 (a very happy person); 2 (relative happiness): “Compared to most of my peers, I consider myself…” 1 (less happy) to 7 (more happy); 3 (optimistic bias): “Some people are generally very happy. They enjoy life regardless of what is going on, getting the most out of everything. To what extent does this characterization describe you?” 1 (not at all) to 7 (a great deal); and 4 (pessimistic bias): “Some people are generally not very happy. Although they are not depressed, they never seem as happy as they might be. To what extent does this characterization describe you?” 1 (not at all) to 7 (a great deal). The internal consistency, test-retest reliability, and convergent discriminant validity of the SHS have previously been confirmed [30,31]. Cronbach’s alphas for the SHS scores were 0.83 and 0.88 in the Japanese and Canadian groups, respectively. Although the average score of the 4 items is typically used to evaluate subjective happiness, in the present study, the 4 individual rating scores were compared between Japan and Canada because each item evaluates a different concept (general happiness, relative happiness, optimistic bias, and pessimistic bias).

### Evaluation of situation-specific happiness

To assess situation-specific happiness, we created an 8-item questionnaire. Based on previous psychological studies [8,10], we selected 8 occasions that may induce a feeling of happiness. The participants were asked to evaluate their happiness on a 5-point Likert scale (1: not happy at all; 2: slightly happy; 3: moderately happy; 4: very happy; 5: extremely happy) when encountering the following 8 situations: (1) When you accomplish a goal (Accomplishment), (2) When you are engaging in something you enjoy (Engagement), (3) When people around you are happy (Being surrounded by happy people), (4) When you are having fun everyday (Fun days), (5) When you have no worries (No worries), (6) When you have good personal relationships (Good personal relationships), (7) When you have good luck (Good luck), and (8) When your financial situation is good (Good financial situation). Cronbach’s alphas for this questionnaire were 0.82 and 0.80 in the Japanese and Canadian groups, respectively. In the present study, we compared both the average combined and individual item scores between Japan and Canada. One Japanese participant skipped items 6 (Good personal relationships) and 7 (Good luck).

### Genotyping

Nail samples were collected, from which genomic DNA was extracted using ISOHAIR kits (NIPPON GENE CO., LTD, Tokyo, Japan). The SNP markers for rs806377 (*CNR1*) were genotyped using TaqMan SNP Genotyping Assays (Thermo Fisher Scientific Inc., Waltham, Massachusetts), which were functionally tested by the manufacturer and available on demand. Each SNP assay contains forward and reverse PCR primers as well as 2 allele-specific probes conjugated with either VIC or FAM fluorescent marker. Each PCR mixture consisted of DNA template, the SNP-specific Genotyping Assay, and Taqman Genotype master mix (Thermo Fisher Scientific Inc.). All PCRs and allelic discrimination reactions were performed on the StepOne Plus Real-Time PCR System (Thermo Fisher Scientific Inc.).

### Statistical analyses

All data analyses were conducted using SPSS version 18 (International Business Machines Corporation [IBM], Armonk, NY). The genotype distributions of *CNR1* were compared between the 2 study groups by Pearson's chi-square test. Because a previous study reported a gene-sex interaction related to happiness [32], subjective happiness levels and situation-specific happiness were analyzed using a 3-way (country [Japan or Canada], sex [male or female], and *CNR1* genotype [CC, CT, or TT]) analysis of variance (ANOVA) followed by Bonferroni-corrected multiple comparisons. In addition, to reveal the association between subjective happiness levels and situational happiness, Pearson’s correlation coefficients were computed, followed by false discovery rate (FDR)-corrected multiple comparisons, typically used on large numbers of tests.

## Results

### Difference in *CNR1* genotype distribution between Japanese and Canadian participants

We first compared the *CNR1* genotype distributions between Japan and Canada. The *CNR1* genotype frequencies in the Japanese individuals were: 24 CC (9.3%), 113 CT (43.6%), and 122 TT (47.1%). Among the Canadian participants, 42 were CC (23.2%), 96 CT (53.0%), and 43 TT (23.8%). The difference in the genotype distribution was significant as assessed by Pearson's chi-square test (*p* = 0.001).

### Main effects of country, sex, and *CNR1* genotype on the subjective happiness level

**Fig 1:**
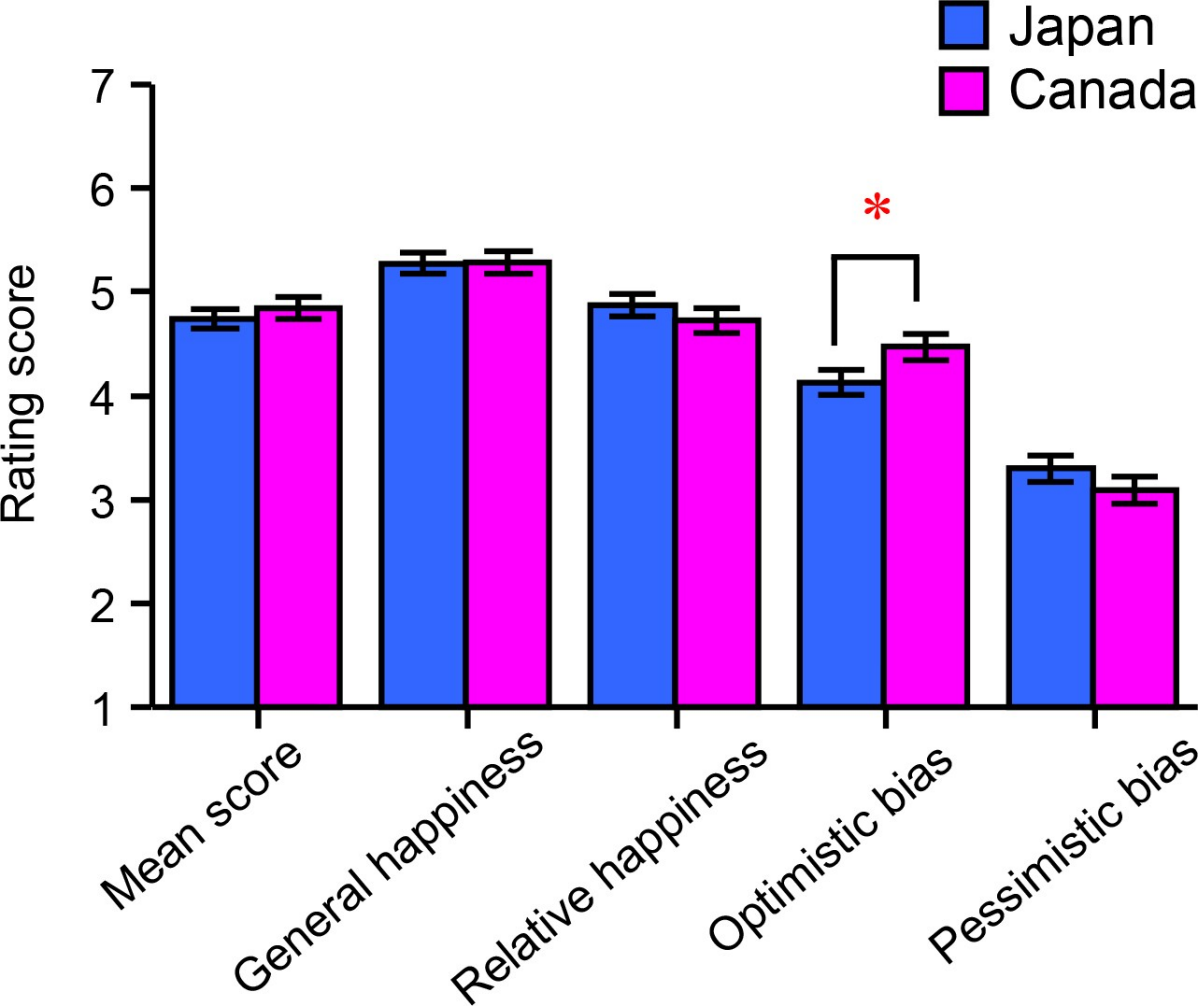
Main effect of country on the subjective happiness level. The bar graph shows the rating scores on each criterion of the Subjective Happiness Scale, including the mean total score (subjective happiness level), general happiness, relative happiness, optimistic bias, and pessimistic bias. Each column and its error bars represent the mean ± standard error of the mean; * *p* < 0.05.

Fig 1 shows the cultural differences in the SHS scores. A 2 (country: Japan, Canada) × 2 (sex: male, female) × 3 (*CNR1* genotype: CC, CT, TT) ANOVA revealed no significant main effects of country, sex, or *CNR1* genotype on the mean combined score. When analyzed for each item, there was a significant main effect of country on the optimistic bias score [*F*(1, 428) = 3.897, *p* = 0.049, *η*^2^_p_ = 0.009], indicating that optimistic bias was significantly higher in the Canadian group than in the Japanese group. S1 Table summarizes the means and standard errors of the mean (SEMs) for each item of the SHS.

### Main effects of country, sex, and *CNR1* genotype on situation-specific happiness

**Fig 2:**
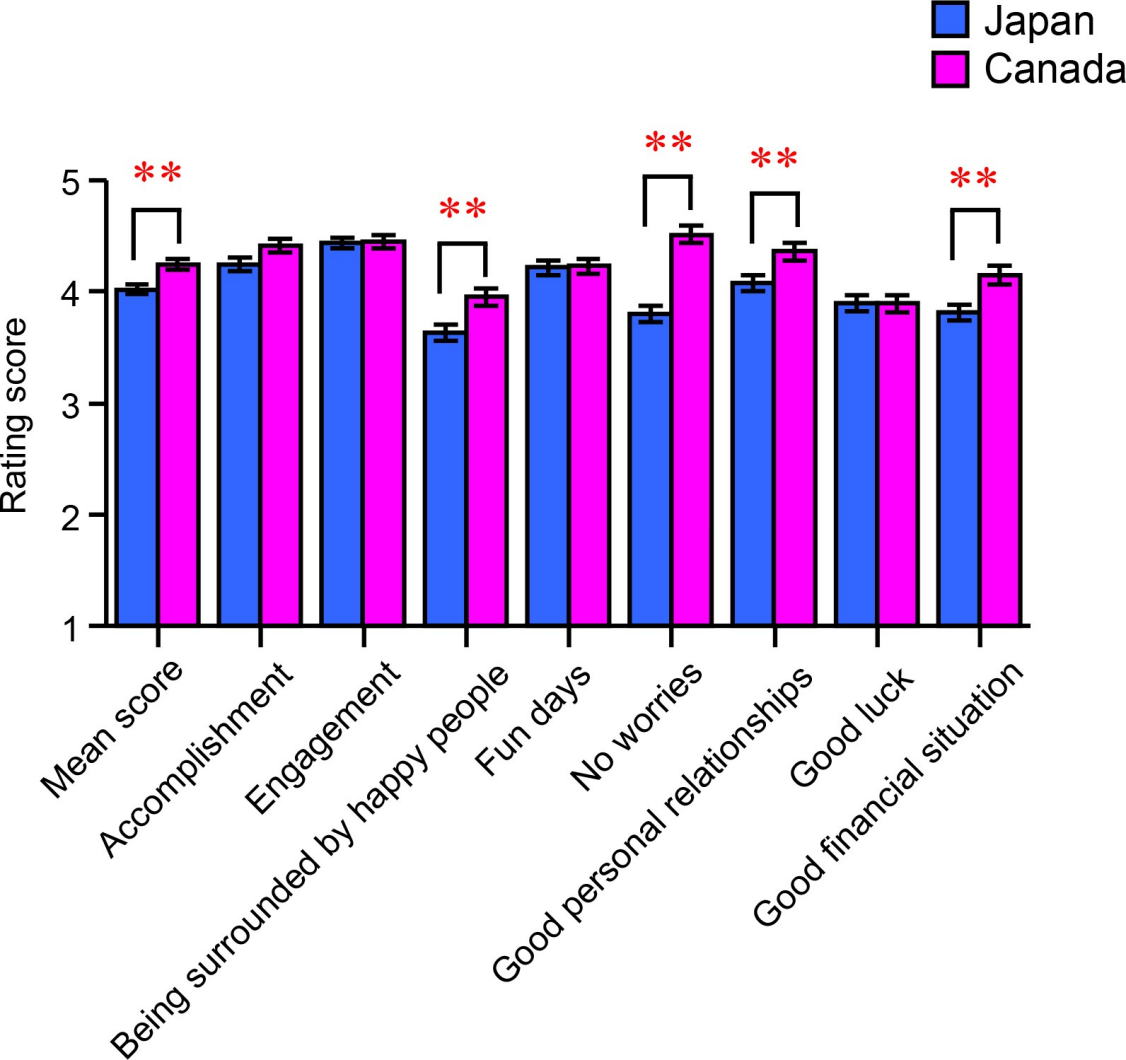
Main effect of country on situation-specific happiness. The bar graph shows the rating scores for each criterion of our original questionnaire. Each column and its error bars represent the mean ± standard error of the mean; ** *p* < 0.01.

Fig 2 shows the cultural differences in situational happiness as assessed by our original 8-item questionnaire. In the present sample, there was a significant main effect of country on the mean combined score [*F*(1, 427) = 11.841, *p* = 0.001, *η*^2^_p_ = 0.027], indicating that Canadians were more likely to be situationally happy than Japanese. When the mean score was analyzed for each item, there were significant main effects of being surrounded by happy people [*F*(1, 427) = 8.913, *p* = 0.003, *η*^2^_p_ = 0.020], having no worries [*F*(1, 428) = 41.822, *p* < 0.001, *η*^2^_p_ = 0.089], good personal relationships [*F*(1, 427) = 7.596, *p* = 0.006, *η*^2^_p_ = 0.017], and good financial situation [*F*(1, 428) = 9.300, *p* = 0.002, *η*^2^_p_ = 0.021]. A multiple-comparisons test indicated that situational happiness owing to being surrounded by happy people (*p* = 0.003), having no worries (*p* < 0.001), maintaining good personal relationships (*p* = 0.006), and being in a good financial situation (*p* = 0.002) was significantly higher in the Canadian sample than in the Japanese group. S2 Table summarizes the means and SEMs for each item.

### Correlation between the subjective happiness level and situational happiness

In the Japanese group, the mean combined SHS score was positively correlated with the mean score of situation-specific happiness [*r*(258) = 0.220, *p* < 0.001], as well as happiness accompanying accomplishment [*r*(259) = 0.269, *p* < 0.001], engagement [*r*(259) = 0.230, *p* < 0.001], being surrounded by happy people [*r*(259) = 0.259, *p* < 0.001], fun days [*r*(259) = 0.160, *p* = 0.010], and good luck [*r*(258) = 0.201, *p* = 0.001]. S3 Table summarizes Pearson’s correlation coefficients for each variable in the Japanese group. In the Canadian group, the mean SHS score was positively correlated with the mean score of situational happiness [*r*(181) = 0.436, *p* < 0.001], as well as happiness accompanying accomplishment [*r*(181) = 0.438, *p* < 0.001], engagement [*r*(181) = 0.359, *p* < 0.001], being surrounded by happy people [*r*(181) = 0.382, *p* < 0.001], fun days [*r*(181) = 0.297, *p* < 0.001], having no worries [*r*(181) = 0.208, *p* = 0.005], good personal relationships [*r*(181) = 0.197, *p* = 0.008], good luck [*r*(181) = 0.208, *p* = 0.005], and good financial situation [*r*(181) = 0.222, *p* = 0.003]. S4 Table summarizes Pearson’s correlation coefficients for each variable in the Canadian group.

### Interaction effect of country and *CNR1* genotype on the subjective happiness level

**Fig 3:**
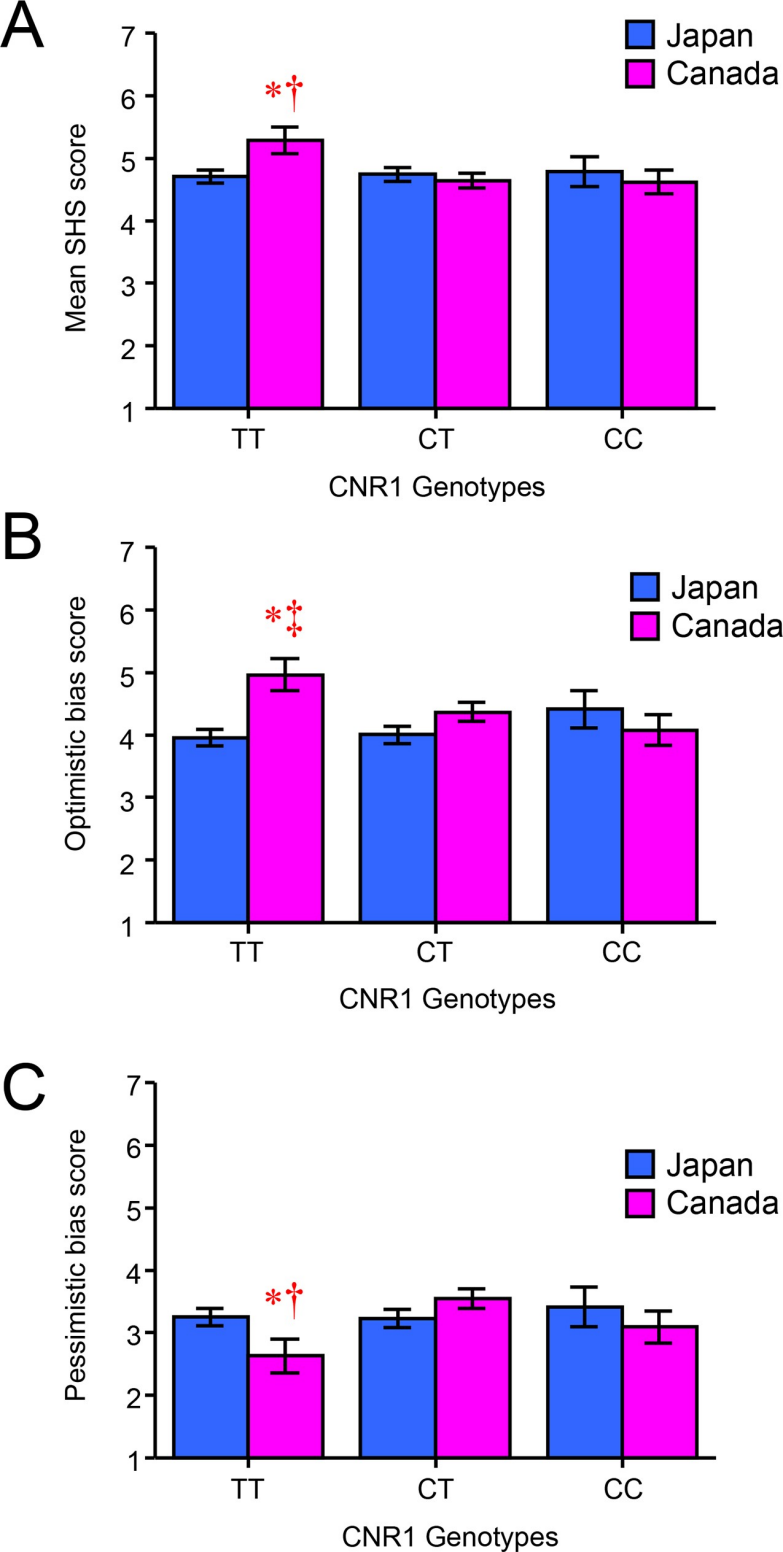
Interaction effect of country and cannabinoid receptor 1 (*CNR1*) genotype on the Subjective Happiness Scale (SHS) score. Each column represents the mean SHS total (A), optimistic bias (B), or pessimistic bias (C) score for each *CNR1* genotype (CC, CT, TT) in the Japan and Canada samples. * *p* < 0.05 vs. TT genotype in Japanese participants. † *p* < 0.05 vs. CT genotype in Canadian participants. ‡ *p* < 0.05 vs. CC genotype in Canadian participants.

Fig 3 shows the effect of *CNR1* genotype on subjective happiness, and Table 1 summarizes the means and SEMs of each item. A 2 (country: Japan, Canada) × 2 (sex: male, female) × 3 (*CNR1* genotype: CC, CT, TT) ANOVA revealed a significant interaction of country and *CNR1* genotype on the mean SHS score [*F*(2, 428) = 3.300, *p* = 0.038, *η*^2^_p_ = 0.015]. In the Japanese cohort, the mean combined SHS score was the highest in individuals with the CC genotype, whereas in the Canadian sample, it was the highest in individuals with the TT genotype. A multiple-comparisons test indicated that the mean score of Canadian individuals with the TT genotype was significantly higher than that of Canadian individuals with the CT genotype (*p* = 0.021), as well as Japanese individuals with the TT genotype (*p* = 0.012). For each SHS criterion, the ANOVA also revealed a significant interaction effect of country and *CNR1* genotype on the optimistic bias score [*F*(2, 428) = 3.986, *p* = 0.019, *η*^2^_p_ = 0.018]. A multiple-comparisons test showed that the individual SHS scores of Canadian participants with the TT genotype were significantly higher than those of Canadian individuals with the CC genotype (*p* = 0.039), as well as Japanese subjects with the TT genotype (*p* = 0.001). The ANOVA also revealed a significant interaction effect of country and *CNR1* genotype on the pessimistic bias score [*F*(2, 428) = 3.397, *p* = 0.034, *η*^2^_p_ = 0.016]. A multiple-comparisons test showed that the individual rating scores of Canadian TT carriers were significantly lower than those of Canadian individuals with the CT genotype (*p* = 0.012) and Japanese subjects with the TT genotype (*p* = 0.045). In addition, there were no significant interaction effects of country, sex, and *CNR1* genotype on the mean combined or individual SHS scores.

**Table 1:** Effects of cannabinoid receptor 1 (*CNR1*) polymorphisms on the Subjective Happiness Scale (SHS) scores in Japanese and Canadian participants.

Variables	Japan			Canada			*p* value
	CC	CT	TT	CC	CT	TT	
**Mean score**	4.786 ± 0.240	4.739 ± 0.109	4.703 ± 0.106	4.617 ± 0.195	4.639 ± 0.119	5.285 ± 0.206	0.038
**General happiness**	5.267 ± 0.245	5.302 ± 0.112	5.260 ± 0.108	4.983 ± 0.199	5.180 ± 0.122	5.688 ± 0.210	0.099
**Relative happiness**	4.878 ± 0.278	4.882 ± 0.127	4.849 ± 0.122	4.500 ± 0.225	4.557 ± 0.138	5.121 ± 0.238	0.154
**Optimistic bias**	4.411 ± 0.301	4.003 ± 0.137	3.953 ± 0.132	4.075 ± 0.244	4.369 ± 0.150	4.959 ± 0.258	0.019
**Pessimistic bias**	3.411 ± 0.320	3.230 ± 0.146	3.249 ± 0.141	3.092 ± 0.259	3.550 ± 0.159	2.629 ± 0.274	0.034

Results are expressed as means ± standard errors of the mean. The variables were compared using a 2 (country: Japan and Canada) x 2 (sex: male and female) x 3 (*CNR1* genotype: CC, CT, and TT) ANOVA; the *p*-value for the country × *CNR1* genotype interaction is shown.

### Interaction effect of country and *CNR1* genotype on situation-specific happiness

**Fig 4:**
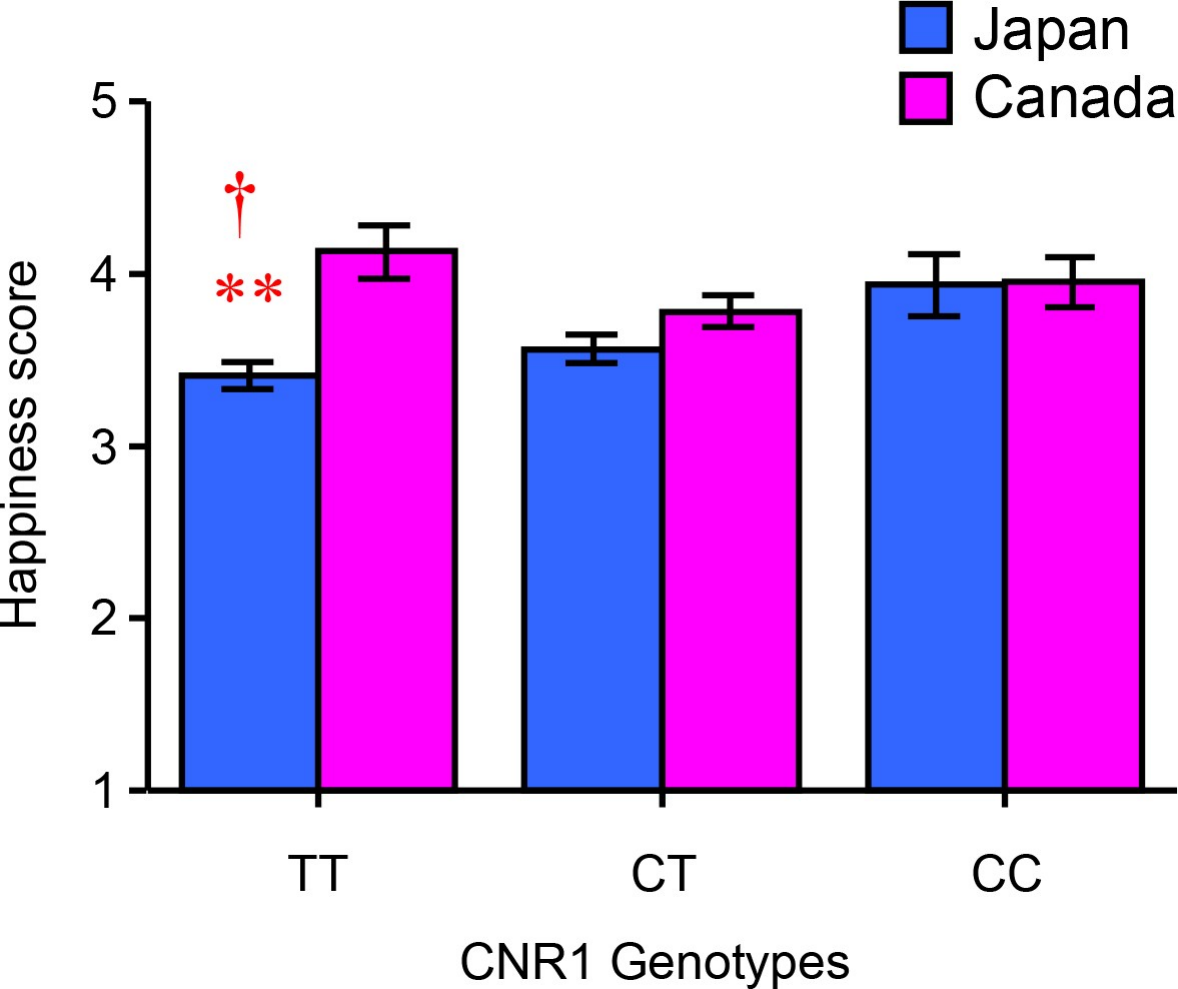
Interaction effect of country and cannabinoid receptor 1 (*CNR1*) genotype on situation-specific happiness. Each column represents the mean score of happiness accompanying being surrounded by happy people for each *CNR1* genotype (CC, CT, TT) in the Japan and Canada samples. ** *p* < 0.01 vs. TT genotype in Canadian participants., † *p* < 0.05 vs. CC genotype in Japanese participants.

The effects of *CNR1* genotype on situation-specific happiness are shown in Fig 4, and the means and SEMs of each item are summarized in Table 2. A 2 (country: Japan, Canada) × 2 (sex: male, female) × 3 (*CNR1* genotype: CC, CT, TT) ANOVA revealed a significant interaction effect of country and *CNR1* genotype on happiness accompanying being surrounded by happy people [*F*(2, 428) = 3.664, *p* = 0.026, *η*^2^_p_ = 0.017]. In the Japanese cohort, the mean score of situational happiness related to being surrounded by happy people was the highest in individuals with the CC genotype, whereas in the Canadian sample, it was the highest in individuals with the TT genotype. A multiple-comparisons test revealed that the mean score of the Japanese TT carriers was significantly lower than those of Japanese individuals with the CC genotype (*p* = 0.028) and Canadian participants with the TT genotype (*p* < 0.001). In addition, there were no significant interaction effects of country, sex, and *CNR1* genotype on happiness accompanying being surrounded by happy people.

**Table 2:** Effects of cannabinoid receptor 1 (*CNR1*) polymorphisms on situation-specific happiness in Japanese and Canadian participants.

Variables	Japan			Canada			*p* value
	CC	CT	TT	CC	CT	TT	
**Mean score**	4.057 ± 0.12	4.071 ± 0.052	3.926 ± 0.049	4.303 ± 0.091	4.213 ± 0.056	4.211 ± 0.096	0.524
**Accomplishment**	4.211 ± 0.149	4.232 ± 0.068	4.289 ± 0.065	4.408 ± 0.120	4.312 ± 0.074	4.512 ± 0.127	0.674
**Engagement**	4.433 ± 0.135	4.417 ± 0.061	4.454 ± 0.059	4.400 ± 0.109	4.357 ± 0.067	4.577 ± 0.116	0.505
**Being surrounded by happy people**	3.933 ± 0.183	3.561 ± 0.083	3.410 ± 0.081	3.950 ± 0.148	3.782 ± 0.091	4.126 ± 0.157	0.026
**Fun days**	4.244 ± 0.161	4.321 ± 0.074	4.082 ± 0.071	4.208 ± 0.131	4.212 ± 0.080	4.262 ± 0.138	0.313
**No worries**	3.878 ± 0.189	3.864 ± 0.086	3.659 ± 0.083	4.608 ± 0.153	4.472 ± 0.094	4.453 ± 0.162	0.687
**Good human relationships**	4.033 ± 0.175	4.253 ± 0.080	3.945 ± 0.077	4.300 ± 0.142	4.420 ± 0.087	4.353 ± 0.150	0.503
**Good luck**	3.933 ± 0.190	3.910 ± 0.087	3.835 ± 0.084	4.100 ± 0.154	3.963 ± 0.095	3.605 ± 0.163	0.333
**Good financial situation**	3.789 ± 0.188	3.923 ± 0.085	3.731 ± 0.082	4.450 ± 0.152	4.188 ± 0.093	3.802 ± 0.161	0.147

Results are expressed as means ± standard errors of the mean. The variables were compared using a 2 (country: Japan and Canada) x 2 (sex: male and female) x 3 (*CNR1* polymorphism: CC, CT, and TT) ANOVA; the *p*-value for the country × *CNR1* polymorphism interaction is shown.

## Discussion

In the present study, we investigated the differences in the subjective happiness level between Japanese and Canadian population samples. The United Nations releases the *World Happiness Report* (http://worldhappiness.report/), which ranks more than 150 countries by their happiness levels using Cantril’s ladder question: “Please imagine a ladder, with steps numbered from 0 at the bottom to 10 at the top. The top of the ladder represents the best possible life for you and the bottom of the ladder represents the worst possible life for you. On which step of the ladder would you say you personally feel you stand at this time?” In the *World Happiness Report 2017*, Norway is at the top of happiness ranking (average ladder score: 7.537). Denmark is in the 2nd place (7.522), Iceland in 3rd (7.504), Switzerland in 4th (7.494), Finland in 5th (7.469), the Netherlands in 6th (7.377), and Canada in 7th (7.316). In contrast, Japan is in the 51st place (5.920), suggesting that it is a relatively unhappy country. However, in the present study, the mean SHS scores were similar between the Japanese and Canadian subjects, in an apparent contradiction with the *World Happiness Report 2017* rankings. This discrepancy may at least partially be explained by the participants’ age: the mean age of our participants was only 19.51 years in Japan and 19.47 years in Canada. The White Paper on the National Lifestyle 2008 by the Cabinet Office of Japan (http://warp.da.ndl.go.jp/info:ndljp/pid/9990748/www5.cao.go.jp/seikatsu/whitepaper/h20/06_eng/index.html) states that the subjective happiness level as a function of age follows a U-curve, and the happiness levels of young Japanese people (under 20 years) is the highest compared to the other ages, including senior years. Thus, the Japanese subjects in the present study represented the group with the highest happiness in Japan, resulting in the mean SHS scores showing no significant difference between the Japanese and Canadian participants. However, the mean rating score of optimistic bias in the Canadian participants was significantly higher than that in the Japanese group. Optimistic illusion is a well-known characteristic of happy people [8]. Because happy people have a high capacity for optimistic illusion, they tend to expect positive outcomes, and thus may maintain high happiness levels for a long time [33–35]. The relatively low positive attitude toward life in Japanese people may be one of the reasons for decreasing global happiness in Japan.

The mean situation-specific happiness score on our questionnaire in the Canadian participants was significantly higher than that in the Japanese individuals. Correlation analysis also indicated that this score was positively correlated with the mean SHS score in both groups. Although there is a conceptual distinction between the subjective happiness level and situation-specific happiness, these 2 aspects of happiness are interrelated [36–38]. Young Japanese people have fewer happy experiences compared to young Canadians. Such low situation-specific happiness in Japanese people may also be one of the reasons for decreasing global happiness in Japan. In addition, situational happiness accompanying being surrounded by happy people, having no worries, good interpersonal relationships, and a good financial situation were significantly higher in the Canadian group than in the Japanese group. Furthermore, the mean combined SHS score in the Japanese group was positively correlated only with happiness accompanying accomplishment, engagement, being surrounded by happy people, fun days, and good luck, whereas that in the Canadian sample was positively correlated with all 8 items. Japanese people may be less likely to have a positive life event that impacts the subjective happiness level compared to Canadians.

Importantly, the present findings indicate gene-culture interactions of *CNR1* genotype both in subjective happiness levels and situation-specific happiness. As mentioned earlier, C allele carriers, who may be sensitive to personal relationships, have a relatively high degree of happiness in Japan. Personal relationships have long been considered to be one of the most important modulators of subjective happiness [39], as we experience many hedonic events through personal relationships [37,40]. A good interpersonal relationship also contributes to the phenomenon of happiness spreading from person to person. Previous studies have demonstrated that individuals who are surrounded by happy people are more likely to experience future increases in their subjective happiness [25,26]. Our finding that Japanese individuals with the CC genotype, who react strongly to others’ smiling, reported a higher degree of happiness related to being surrounded by happy people than did those with the TT genotype stands to reason. In contrast, in the Canadian group, individuals with the TT genotype had a higher level of subjective happiness (high mean SHS score, high optimistic bias, and low pessimistic bias) compared to that in C carries, although there was no significant difference in the mean SHS score between Canadian individuals with the TT genotype and Canadian individuals with the CC genotype (*p* = 0.056) due to a large variation. This result indicated that the CC genotype may be disadvantageous to subjective happiness in Canada, where the subjective happiness is informed by inner feelings. In Japan, the concept of happiness is mainly linked to fortunate circumstances, whereas it is primarily associated with positive inner feelings in the United States, Canada, Spain, Argentina, Ecuador, and many other countries [9]. Thus, people amidst happy circumstances and opportunities, including happy people, will be happier in Japanese cultures. In contrast, in American cultures, people who are not influenced by others’ well-being may be happier. As aforementioned, although the mechanisms underlying this phenomenon may be complicated, the interaction of social comparison and the social sharing of happiness may be an important contributing factor [17,21–26].

Recent studies provide some insight into the potential biological mechanisms underlying the international differences in happiness. Carriers of the 5HTTLPR L allele, associated with increased serotonin reuptake activity, report significantly higher levels of life satisfaction and happiness compared to S allele carriers [41,42]. The US reports a relatively high level of happiness (14th in the ranking of *World Happiness Report 2017*), and previous findings have indicated that the 5HTTLPR genotype distribution is different in the US (SS: 26%, SL: 49%, LL: 25%) [43] than in Japan (SS: 57.8%, SL: 37.8%, LL: 4.4%) [32]. Thus, cultural differences in happiness might be partly explained by ethnogeographic differences in the distribution of a happiness-related gene polymorphism. A previous study found that the proportion of people with the *CNR1* CC genotype was small in Japan (CC: 10.6%, CT: 43.4%, TT: 46.0%) [7]. In contrast, a study of subjects from several European countries (Germany, the UK, Ireland, and France) reported a much higher frequency of the *CNR1* CC genotype (CC: 30.2%, CT: 48.2%, TT: 21.6%) [44]. The present study confirmed the previously published *CNR1* genotype distribution in the Japanese [7], and found young Japanese individuals with the CC genotype to have a relatively high degree of happiness, if only representing a small fraction of the population. The low frequency of the *CNR1* CC genotype may be another reason for decreasing global happiness in Japan. Thus, the ethnogeographic differences in the *CNR1* genotype frequencies may be closely related to cultural differences in happiness.

In order to strengthen the gene-culture interactions on the perception of happiness, it may be more reasonable to have recruited Japanese ancestry samples both from Japan and Canada or other countries with cultures different from Japan. However, in a previous study, although gene (5HTTLPR)-culture (Japanese and American) interaction was observed, they did not demonstrate significant differences in behavioral data between Asian Americans who were born and raised in the United States and then exposed to East Asian cultures (i.e., China, Korea, Japan, and Taiwan) and European Americans who were born and raised in the United States [45]. Thus, it is possible that people with certain polymorphisms acquire a predominant psychological tendency in the cultural environment, rather than individual differences in genetic polymorphisms that are directly linked to some psychological tendency.

Our study has several limitations. First, we only compared individuals from Japan and Canada, making any generalizations on differences in the effects of *CNR1* polymorphism between different cultures highly speculative. Second, because our goal was to study the interaction between culture and genes, and because the sample size was small, we did not focus on the effects of interactions among genes, culture, or sex on happiness. It is possible that the limited samples may have led to a false conclusion; further studies using larger samples may be required. Furthermore, a multiple-comparisons test showed that overall situational happiness of Japanese female individuals with the TT genotype was significantly higher than that of Japanese male individuals with the same genotype, whereas Canadian female participants with the CT genotype showed significantly higher situation-specific happiness than male CT carriers (data not shown). Because there are sex differences in happiness [46], future studies need to clarify the effects of the interaction among genes, culture, and sex on happiness. Third, polymorphisms in the gene encoding fatty acid amide hydrolase (FAAH), which breaks down anandamide, has recently been associated with happiness as well [47]. Although we only investigated the association between *CNR1* polymorphism and happiness in the present study, it is possible that *FAAH* and cannabinoid receptor gene polymorphisms interact to influence the perception of happiness. Furthermore, we could not conclude whether the *CNR1* genotype was the principle component in the association between gene polymorphisms and the perception of happiness because we did not genotype the samples with a microarray. In fact, a previous genome-wide association study points out the association between gene polymorphisms, other than *CNR1*, and subjective well-being [48]. The effects of such gene-gene interactions on happiness require further study. Fourth, the present study did not consider the possibility of the influence of other variables on happiness perception. For example, socioeconomic status (SES) influence the perception of happiness [49], and a previous study indicated that SES may modulate the effect of genetic polymorphisms, such as 5HTTLPR, on emotional behaviors [50]. Thus, it is possible that the SES may influence the current findings. Fifth, we assumed that the influence of rs806377 on CNR1 expression level is the same in Japan and Canada. However, it is possible that the causal SNP in regulating *CNR1* would be different between Canadians and Japanese. Rs806377 is an intronic SNP of *CNR1*, not coding SNP or regulatory SNP [51], and the present results indicated that the *CNR1* genotype distributions differed between Japan and Canada, suggesting that there is a possibility that there is a difference in the structure of the intron region. Thus, further studies focusing the influence of rs806377 on CNR1 expression level between Japanese and Canadians may be required.

Nevertheless, our results provide a plausible explanation of how *CNR1* polymorphism may have different effects on happiness in different cultures. If corroborated, our findings can be used to develop strategies to increase the degree of happiness tailored to specific countries or groups of countries, in a major contribution to the field of social psychology.

## Supporting information

S1 Table Main effects of country, sex, and cannabinoid receptor 1 (*CNR1*) genotype on the Subjective Happiness Scale score. Results are expressed as means ± standard errors of the mean. Variables were compared using a 2 (country: Japan, Canada) × 2 (sex: male, female) × 3 (*CNR1* genotype: CC, CT, TT) ANOVA followed by Bonferroni-corrected multiple comparisons. No significant main effects of sex and *CNR1* genotype were evident for any study variables.
(DOCX)

S2 Table Main effects of country, sex, and cannabinoid receptor 1 (*CNR1*) genotype on situation-specific happiness. Results are expressed as means ± standard errors of the mean. The variables were compared using a 2 (country: Japan, Canada) × 2 (sex: male, female) × 3 (*CNR1* genotype: CC, CT, TT) ANOVA, followed by Bonferroni-corrected multiple comparisons. Sex showed a significant main effect on the mean combined score [*F*(1, 427) = 6.496, *p* = 0.011, *η*^2^_p_ = 0.015], with the mean score in women being significantly higher than that in men (*p* = 0.011). When analyzed for each item, significant main effects of sex on happiness accompanying fun days [*F*(1, 428) = 4.119, *p* = 0.043, *η*^2^_p_ = 0.010], good personal relationships [*F*(1, 427) = 8.901, *p* = 0.003, *η*^2^_p_ = 0.020], and good financial situation [*F*(1, 428) = 5.743, *p* = 0.017, *η*^2^_p_ = 0.013] were observed. *CNR1* genotype demonstrated a significant main effect on situational happiness accompanying good financial situation [*F*(2, 428) = 4.173, *p* = 0.016, *η*^2^_p_ = 0.019], and a multiple-comparisons test indicated that this factor made CT genotype carriers significantly happier than TT genotype carriers (*p* = 0.027).
(DOCX)

S3 Table Correlations between the Subjective Happiness Scale (SHS) scores and situation-specific happiness in Japanese participants. The table shows Pearson’s correlation coefficients. Asterisks indicate statistically significant correlations after false discovery rate correction. For each item of the SHS, the rating score of general happiness was positively correlated with the mean score of situational happiness [*r*(258) = 0.258, *p* < 0.001], as well as happiness related to accomplishment [*r*(259) = 0.272, *p* < 0.001], engagement [*r*(259) = 0.248, *p* < 0.001], being surrounded by happy people [*r*(259) = 0.266, *p* < 0.001], fun days [*r*(259) = 0.152, *p* = 0.014], good luck [*r*(258) = 0.166, *p* = 0.007], and good financial situation [*r*(259) = 0.164, *p* = 0.008]. The rating score of relative happiness was positively correlated with the mean score of situational happiness [*r*(258) = 0.229, *p* < 0.001], as well as happiness accompanying accomplishment [*r*(259) = 0.245, *p* < 0.001], engagement [*r*(259) = 0.266, *p* < 0.001], being surrounded by happy people [*r*(259) = 0.239, *p* < 0.001], and good luck [*r*(258) = 0.192, *p* = 0.002]. The rating score of optimistic bias showed a positive correlation with the mean score of situation-specific happiness [*r*(258) = 0.136, *p* = 0.029] and happiness related to accomplishment [*r*(259) = 0.186, *p* = 0.003], being surrounded by happy people [*r*(259) = 0.204, *p* = 0.001], fun days [*r*(259) = 0.152, *p* = 0.015], and good luck [*r*(258) = 0.144, *p* = 0.021]. The pessimistic bias score was negatively correlated with happiness accompanying accomplishment [*r*(259) = −0.198, *p* = 0.001], engagement [*r*(259) = −0.160, *p* = 0.010], being surrounded by happy people [*r*(259) = −0.161, *p* = 0.009], and good luck [*r*(258) = −0.164, *p* = 0.008].
(DOCX)

S4 Table Correlations between the Subjective Happiness Scale (SHS) scores and situation-specific happiness in Canadian participants. The table shows Pearson’s correlation coefficients. Asterisks indicate statistically significant correlations after false discovery rate correction. For each item of the SHS, the rating score of general happiness was positively correlated with the mean score of situation-specific happiness [*r*(181) = 0.419, *p* < 0.001], as well as happiness accompanying accomplishment [*r*(181) = 0.407, *p* < 0.001], engagement [*r*(181) = 0.449, *p* < 0.001], being surrounded by happy people [*r*(181) = 0.318, *p* < 0.001], fun days [*r*(181) = 0.301, *p* < 0.001], having no worries [*r*(181) = 0.258, *p* < 0.001], good personal relationships [*r*(181) = 0.185, *p* = 0.013], and good financial situation [*r*(181) = 0.217, *p* = 0.003]. The rating score of relative happiness showed a positive correlation with the mean score of situational happiness [*r*(181) = 0.402, *p* < 0.001], as well as happiness accompanying accomplishment [*r*(181) = 0.376, *p* < 0.001], engagement [*r*(181) = 0.290, *p* < 0.001], being surrounded by happy people [*r*(181) = 0.346, *p* < 0.001], funny days [*r*(181) = 0.263, *p* < 0.001], no worries [*r*(181) = 0.180, *p* = 0.015], good personal relationships [*r*(181) = 0.175, *p* = 0.018], good luck [*r*(181) = 0.221, *p* = 0.003], and good financial situation [*r*(181) = 0.252, *p* = 0.001]. Optimistic bias was positively correlated with the mean score of situational happiness [*r*(181) = 0.344, *p* < 0.001], and happiness accompanying accomplishment [*r*(181) = 0.367, *p* < 0.001], engagement [*r*(181) = 0.224, *p* = 0.002], being surrounded by happy people [*r*(181) = 0.359, *p* < 0.001], fun days [*r*(181) = 0.226, *p* = 0.002], good personal relationships [*r*(181) = 0.178, *p* = 0.016], and good luck [*r*(181) = 0.188, *p* = 0.011]. Pessimistic bias was negatively correlated with the mean score of situation-specific happiness [*r*(181) = −0.349, *p* < 0.001], as well as happiness related to accomplishment [*r*(181) = −0.368, *p* < 0.001], engagement [*r*(181) = −0.299, *p* < 0.001], being surrounded by happy people [*r*(181) = −0.296, *p* < 0.001], fun days [*r*(181) = −0.243, *p* = 0.001], good luck [*r*(181) = −0.176, *p* = 0.018], and good financial situation [*r*(181) = −0.173, *p* = 0.020].
(DOCX)
