# Supplementary material for: Culture and cannabinoid receptor gene polymorphism interact to influence the perception of happiness
Source: PLoS One. 2018 Dec 21;13(12):e0209552. doi: 10.1371/journal.pone.0209552 (PMC6303049; doi:10.1371/journal.pone.0209552)
Supplement: S1 Table — Results are expressed as means ± standard errors of the mean. Variables were compared using a 2 (country: Japan, Canada) × 2 (sex: male, female) × 3 (CNR1 genotype: CC, CT, TT) ANOVA followed by Bonferroni-corrected multiple comparisons. No significant main effects of sex and CNR1 genotype were evident for any study variables. (DOCX) [file pone.0209552.s001.docx]

|  | **Country** | | **Sex** | | ***CNR1* genotype** | | |
| --- | --- | --- | --- | --- | --- | --- | --- |
| **Variable** | **Japan** | **Canada** | **Men** | **Women** | **CC** | **CT** | **TT** |
| **Mean score** | 4.743 ± 0.095 | 4.847 ± 0.102 | 4.766 ± 0.115 | 4.823 ± 0.080 | 4.701 ± 0.155 | 4.689 ± 0.081 | 4.994 ± 0.116 |
| **General happiness** | 5.276 ± 0.097 | 5.284 ± 0.105 | 5.189 ± 0.117 | 5.371 ± 0.081 | 5.125 ± 0.158 | 5.241 ± 0.083 | 5.474 ± 0.118 |
| **Relative happiness** | 4.869 ± 0.110 | 4.726 ± 0.119 | 4.682 ± 0.133 | 4.913 ± 0.092 | 4.689 ± 0.179 | 4.719 ± 0.094 | 4.985 ± 0.134 |
| **Optimistic bias** | 4.123 ± 0.119 | 4.468 ± 0.128 | 4.340 ± 0.144 | 4.250 ± 0.100 | 4.243 ± 0.194 | 4.186 ± 0.102 | 4.456 ± 0.145 |
| **Pessimistic bias** | 3.297 ± 0.126 | 3.090 ± 0.136 | 3.146 ± 0.153 | 3.241 ± 0.106 | 3.251 ± 0.206 | 3.390 ± 0.108 | 2.939 ± 0.154 |
