# Supplementary material for: Culture and cannabinoid receptor gene polymorphism interact to influence the perception of happiness
Source: PLoS One. 2018 Dec 21;13(12):e0209552. doi: 10.1371/journal.pone.0209552 (PMC6303049; doi:10.1371/journal.pone.0209552)
Supplement: S2 Table — Results are expressed as means ± standard errors of the mean. The variables were compared using a 2 (country: Japan, Canada) × 2 (sex: male, female) × 3 (CNR1 genotype: CC, CT, TT) ANOVA, followed by Bonferroni-corrected multiple comparisons. Sex showed a significant main effect on the mean combined score [F(1, 427) = 6.496, p = 0.011, η2p = 0.015], with the mean score in women being significantly higher than that in men (p = 0.011). When analyzed for each item, significant main effects of sex on happiness accompanying fun days [F(1, 428) = 4.119, p = 0.043, η2p = 0.010], good personal relationships [F(1, 427) = 8.901, p = 0.003, η2p = 0.020], and good financial situation [F(1, 428) = 5.743, p = 0.017, η2p = 0.013] were observed. CNR1 genotype demonstrated a significant main effect on situational happiness accompanying good financial situation [F(2, 428) = 4.173, p = 0.016, η2p = 0.019], and a multiple-comparisons test indicated that this factor made CT genotype carriers significantly happier than TT genotype carriers (p = 0.027). (DOCX) [file pone.0209552.s002.docx]

|  | **Country** | | **Sex** | | ***CNR1* genotype** | | |
| --- | --- | --- | --- | --- | --- | --- | --- |
| **Variable** | **Japan** | **Canada** | **Men** | **Women** | **CC** | **CT** | **TT** |
| **Mean score** | 4.018 ± 0.044 | 4.243 ± 0.048 | 4.047 ± 0.054 | 4.213 ± 0.037 | 4.180 ± 0.072 | 4.142 ± 0.038 | 4.068 ± 0.054 |
| **Accomplishment** | 4.244 ± 0.059 | 4.411 ± 0.063 | 4.270 ± 0.071 | 4.385 ± 0.1049 | 4.310 ± 0.096 | 4.272 ± 0.050 | 4.401 ± 0.071 |
| **Engagement** | 4.435 ± 0.053 | 4.445 ± 0.058 | 4.410 ± 0.064 | 4.470 ± 0.045 | 4.417 ± 0.087 | 4.387 ± 0.046 | 4.515 ± 0.065 |
| **Being surrounded by happy people** | 3.635 ± 0.072 | 3.952 ± 0.078 | 3.754 ± 0.087 | 3.834 ± 0.061 | 3.942 ± 0.118 | 3.671 ± 0.062 | 3.768 ± 0.088 |
| **Fun days** | 4.216 ± 0.064 | 4.227 ± 0.069 | 4.127 ± 0.077 | 4.317 ± 0.053 | 4.226 ± 0.104 | 4.266 ± 0.054 | 4.172 ± 0.078 |
| **No worries** | 3.800 ± 0.075 | 4.511 ± 0.081 | 4.089 ± 0.090 | 4.222 ± 0.063 | 4.243 ± 0.122 | 4.168 ± 0.064 | 4.056 ± 0.091 |
| **Good personal relationships** | 4.077 ± 0.069 | 4.358 ± 0.075 | 4.065 ± 0.084 | 4.369 ± 0.058 | 4.167 ± 0.113 | 4.336 ± 0.059 | 4.149 ± 0.084 |
| **Good luck** | 3.893 ± 0.075 | 3.889 ± 0.081 | 3.783 ± 0.091 | 4.000 ± 0.063 | 4.017 ± 0.123 | 3.937 ± 0.064 | 3.720 ± 0.092 |
| **Good financial situation** | 3.814 ± 0.074 | 4.147 ± 0.080 | 3.850 ± 0.090 | 4.111 ± 0.062 | 4.119 ± 0.121 | 4.055 ± 0.063 | 3.766 ± 0.090 |
