# Supplementary material for: Culture and cannabinoid receptor gene polymorphism interact to influence the perception of happiness
Source: PLoS One. 2018 Dec 21;13(12):e0209552. doi: 10.1371/journal.pone.0209552 (PMC6303049; doi:10.1371/journal.pone.0209552)
Supplement: S3 Table — The table shows Pearson’s correlation coefficients. Asterisks indicate statistically significant correlations after false discovery rate correction. For each item of the SHS, the rating score of general happiness was positively correlated with the mean score of situational happiness [r(258) = 0.258, p < 0.001], as well as happiness related to accomplishment [r(259) = 0.272, p < 0.001], engagement [r(259) = 0.248, p < 0.001], being surrounded by happy people [r(259) = 0.266, p < 0.001], fun days [r(259) = 0.152, p = 0.014], good luck [r(258) = 0.166, p = 0.007], and good financial situation [r(259) = 0.164, p = 0.008]. The rating score of relative happiness was positively correlated with the mean score of situational happiness [r(258) = 0.229, p < 0.001], as well as happiness accompanying accomplishment [r(259) = 0.245, p < 0.001], engagement [r(259) = 0.266, p < 0.001], being surrounded by happy people [r(259) = 0.239, p < 0.001], and good luck [r(258) = 0.192, p = 0.002]. The rating score of optimistic bias showed a positive correlation with the mean score of situation-specific happiness [r(258) = 0.136, p = 0.029] and happiness related to accomplishment [r(259) = 0.186, p = 0.003], being surrounded by happy people [r(259) = 0.204, p = 0.001], fun days [r(259) = 0.152, p = 0.015], and good luck [r(258) = 0.144, p = 0.021]. The pessimistic bias score was negatively correlated with happiness accompanying accomplishment [r(259) = −0.198, p = 0.001], engagement [r(259) = −0.160, p = 0.010], being surrounded by happy people [r(259) = −0.161, p = 0.009], and good luck [r(258) = −0.164, p = 0.008]. (DOCX) [file pone.0209552.s003.docx]

|  | **SHS** | | | | |
| --- | --- | --- | --- | --- | --- |
| **Situations** | **Mean score** | **General happiness** | **Relative happiness** | **Optimistic bias** | **Pessimistic bias** |
| Mean score | 0.220* | 0.258* | 0.229* | 0.136* | −0.128 |
| Accomplishment | 0.269* | 0.272* | 0.245* | 0.186* | −0.198* |
| Engagement | 0.230* | 0.248* | 0.266* | 0.108 | −0.160* |
| Being surrounded by happy people | 0.259* | 0.266* | 0.239* | 0.204* | −0.161* |
| Fun days | 0.160* | 0.152* | 0.131 | 0.152* | −0.097 |
| No worries | −0.025 | 0.071 | 0.040 | −0.080 | 0.081 |
| Good personal relationships | 0.100 | 0.128 | 0.096 | 0.071 | −0.046 |
| Good luck | 0.201* | 0.166* | 0.192* | 0.144* | −0.164* |
| Good financial situation | 0.077 | 0.164* | 0.103 | −0.005 | −0.022 |
